# Simulated effects of nitrogen saturation on the global carbon budget using the IBIS model

**DOI:** 10.1038/srep39173

**Published:** 2016-12-14

**Authors:** Xuehe Lu, Hong Jiang, Jinxun Liu, Xiuying Zhang, Jiaxin Jin, Qiuan Zhu, Zhen Zhang, Changhui Peng

**Affiliations:** 1 Jiangsu Provincial Key Laboratory of Geographic Information Science and Technology, Xianlin Avenue 163, Nanjing 210093, China; 2 International Institute for Earth System Science, Nanjing University, Xianlin Avenue 163, Nanjing 210093, China; 3 USGS Western Geographic Science Center, Menlo Park, CA, 94025, USA; 4 State Key Laboratory of Soil Erosion and Dryland Farming on the Loess Plateau, Northwest A&F University, Yangling 712100, China

## Abstract

Over the past 100 years, human activity has greatly changed the rate of atmospheric N (nitrogen) deposition in terrestrial ecosystems, resulting in N saturation in some regions of the world. The contribution of N saturation to the global carbon budget remains uncertain due to the complicated nature of C-N (carbon-nitrogen) interactions and diverse geography. Although N deposition is included in most terrestrial ecosystem models, the effect of N saturation is frequently overlooked. In this study, the IBIS (Integrated BIosphere Simulator) was used to simulate the global-scale effects of N saturation during the period 1961–2009. The results of this model indicate that N saturation reduced global NPP (Net Primary Productivity) and NEP (Net Ecosystem Productivity) by 0.26 and 0.03 Pg C yr^−1^, respectively. The negative effects of N saturation on carbon sequestration occurred primarily in temperate forests and grasslands. In response to elevated CO_2_ levels, global N turnover slowed due to increased biomass growth, resulting in a decline in soil mineral N. These changes in N cycling reduced the impact of N saturation on the global carbon budget. However, elevated N deposition in certain regions may further alter N saturation and C-N coupling.

Reactive N (nitrogen) in soil is the primary nutrient source for vegetation growth^1^ and exerts a profound influence on the ecosystem’s C (carbon) cycle^2^. Researchers have found that N limitations affect the NPP (Net Primary Productivity) of ecosystems around the globe^3^,^4^. Together, elevated CO_2_ levels and climate change have exacerbated this limitation^5^,^6^. Even in N-rich tropical forests, N availability is a key regulator of C balance^7^. Based on modelling studies, global N limitation reduced C accumulation on land from 19 to 162 Pg C (Pg = 10^15^ g) between the pre-industrial period and the early 21^st^ century^4^,^8^,^9^.

However, the global pattern of N limitation might be altered by N deposition^10^,^11^. With enhanced human activity (e.g., fossil fuel combustion and N fertilization), N deposition has increased from less than 1 Tg N yr^−1^ (Tg = 10^12^ g) in the 1860 s to 25 Tg N yr^−1^ in 2000^12^,^13^ and will likely double over the next 25 years^14^. When N input to the ecosystem exceeds the demands of plants and microbial organisms, N saturation occurs, resulting in a series of changes in several processes, such as N mineralization, nitrification, nitrate leaching and C sequestration^15^. Experiments in Europe and North America have shown that if N deposition is 2.5–3.0 g m^−2^ yr^−1^, N saturation will occur^16^,^17^. In southern China, researchers have found that high levels of N deposition (3.6–3.8 g m^−2^ yr^−1^) led to N saturation in subtropical mature forests^18^. N saturation has been associated with anthropogenic N deposition^19^,^20^. In southern China, N deposition increased from 2.6 to 6.5 g N m^−2^ yr^−1^ along an urban-rural transect, resulting in N saturation in urban and suburban forests^21^.

The effects of N saturation on ecosystems are complex. Researchers have found that in N-saturated ecosystems, N addition does not increase foliar N^22^ which results in the reduction of plant photosynthesis^18^,^23^. In addition, N saturation can decrease C allocation to both leaves and wood^24^, restrict soil respiration^25^,^26^, reduce microbial biomass^27^ and increase N leaching^28^. However, some studies found that N addition continued to result in increased C allocation to aboveground biomass despite saturated soil N^29^. Furthermore, increasing of N leaching is the first, not last, variable to respond to N addition which is different from the statement in N saturation hypotheses^20^. The complexity of these N saturation effects may be due to the spatial and temporal limitations of different research approaches (e.g., long-term observational, gradient, and experimental studies)^30^.

Ecosystem models are suitable for assessing the effects of N addition on ecosystems on large spatial and long temporal scales. Many existing C-N (carbon-nitrogen) coupled models have been used to evaluate global C budgets^4^,^8^,^31^,^32^,^33^,^34^. Modelling has revealed that increased N deposition facilitated the absorption of an additional 0.3–1.3 Pg C yr^−1^ by terrestrial ecosystems in the 1990 s under elevated CO_2_ levels^35^. In the future, elevated N deposition will likely promote the uptake of an extra 0.81 Pg C yr^−1^ by forests^11^. However, the process of N saturation has not been considered in large-scale modelling studies.

Based on the available N saturation literature, we modified a process-based terrestrial ecosystem model — IBIS (the Integrated BIosphere Simulator) - to test the influence of N saturation on ecosystem photosynthesis, C allocation and litter decomposition. Historical N deposition and climate change data were used to drive model simulations. Model experiments were performed to examine the responses of ecosystems to enhanced N deposition and to evaluate the negative effects of N saturation on global C budgets under elevated CO_2_ conditions.

## Results

### The global pattern of N saturation

**Figure 1:**
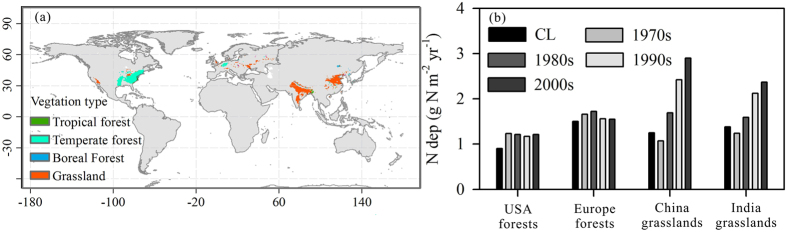
Global N-saturation regions. (**a**) shows distribution of N-saturation regions; (**b**) shows the average N deposition of the main N saturated regions in different time periods. The maps were generated using ArcGIS 10.0 software (https://www.arcgis.com/) and SigmaPlot version 12.0, from Systat Software, Inc., San Jose California USA (https://www.systatsoftware.com).

In our study, N deposition in excess of the N critical load is considered N saturation. The details of model modification, parameterization and determination of the spatial distribution of N critical are introduced in Methods. The spatial distribution of current N saturation is shown in Fig. 1a. Regions of N saturation are mainly located in the US, Europe, India and China, whereas other areas exhibit varying degrees of N deficits. The vegetation in saturated regions is mainly grasslands and temperate forests. N-saturated grasslands are located mainly in India and China, with small regions scattered throughout Europe. N-saturated forests are located in the eastern US and in Europe. N deposition in the saturation regions is high due to fossil fuel combustion and agricultural fertilization^36^.

The multi-year average N deposition for different vegetation types in saturated regions is shown in Fig. 1b. N deposition always exceeded the critical load in US temperate forests, whereas in European forests, N deposition decreased between 1970 and 2009, and current N deposition only marginally exceeds the N critical load. More temperate forests in Europe were located in N-saturated regions in the 1980 s, when N deposition was at its highest value of the past 40 years. In China and India, due to intensified human activity, rapid increases in N deposition resulted in N-saturated grasslands. In the 2000 s, N deposition far exceeded the N critical loads, indicating that the ecosystem was significantly affected by N saturation in N-saturated grassland in China and India.

### Effects of N deposition on the global C budget

Several different simulation scenarios were used to evaluate the effects of N deposition on global C budgets. The details of these simulation scenarios are listed in Table 1. The historical changes of global average NPP and NEP (Net Ecosystem Productivity) were simulated by the NCC (Nitrogen CO_2_ Climate) scenario, and the results are shown in Figure S5. The average NPP was 51.3 Pg C yr^−1^ in the 1970 s and 55.9 Pg C yr^−1^ in the 2000 s, whereas the average NEP was 2.2 Pg C yr^−1^ in the 1970 s and 2.8 Pg C yr^−1^ in the 2000 s. Due to climate change, increasing CO_2_, and elevated N deposition, NPP and NEP increased by 9.0% and 27.2%, respectively, over the past 40 years (Figure S5).

**Figure 2:**
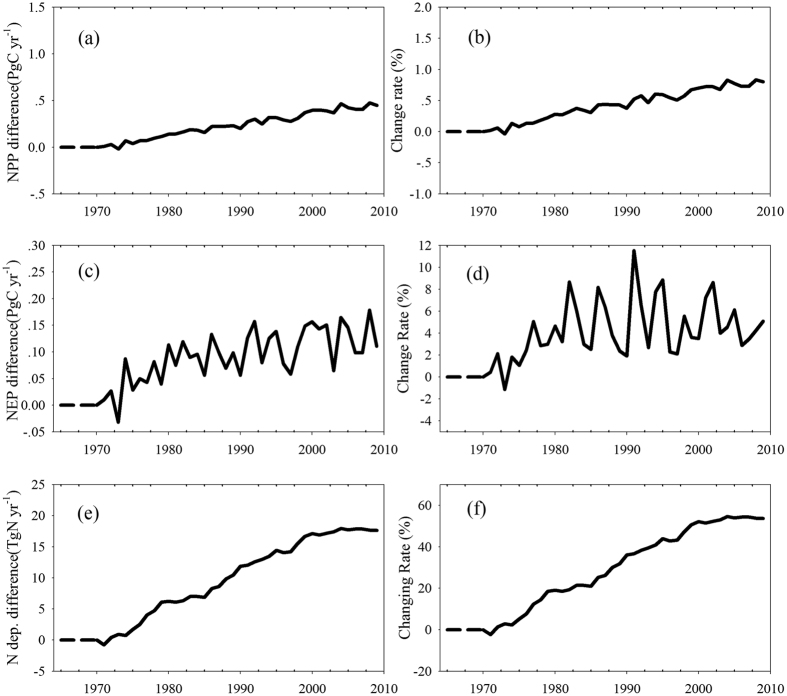
The differences in C budget between simulation scenarios NCC and NNC. (**a**) and (**b**) show NPP difference and change percentage; (**c**) and (**d**) show NEP difference and change percentage; (**e**) and (**f**) show N deposition difference and change percentage.

The effects of N deposition on global C budgets were evaluated by comparing the NNC (No N deposition Change) and NCC scenarios. The results show that N deposition had positive effects on the global C budget over the past few decades. Increased N deposition increased the NPP by 0.23 Pg C yr^−1^ on average (Fig. 2a), which accounts for approximately 0.44% of the global average NPP (Fig. 2b). N deposition promoted an increase in NEP by 0.09 Pg C yr^−1^ on average (Fig. 2c), which corresponds to 4.2% of the global average NEP (Fig. 2d).

Over the past 40 years, the total N deposition increased by 53% (Fig. 2e and f), although it did not result in significant increases in global NPP and NEP. The maximum effects of N deposition were observed in the 2000 s, with NPP increasing by 0.8% and NEP increasing by 6.0%. In general, global C assimilation is limited by N availability. However, increased N addition into ecosystems did not result in a significant increase in C assimilation. The imbalanced spatial distribution of N deposition may be the main reason underlying the contrast between terrestrial ecosystem C assimilation and rapidly increasing N deposition.

The distribution of N deposition is uneven across ecosystems. In this study, we used ecosystem biomes to examine the change in N deposition in different ecosystems. The ecosystem biomes were defined by Roy *et al*.^37^, and the global pattern of this dataset was shown by Beer *et al*.^38^. In this dataset, the cropland area was taken from the MODIS (MODerate resolution Imaging Spectroradiometer) land cover map^39^. As a single biome, croplands accounted for the largest fraction of the global total N deposition (28.7%) in the 2000 s. During the same period, the forest biomes together accounted for approximately 38.4% of the global total N deposition; tropical, temperate, and boreal forests accounted for 18.0%, 14.9% and 5.5%, respectively (Table S4). The increase in the rate of N deposition was significantly different among biomes (Table S4), as was the change in N deposition. N deposition increased rapidly in tropical forest, accounting for approximately 25.9% of the increase in the global total N deposition. Temperate forest biomes also increased markedly, accounting for 11.3% of the increase in global total N deposition. However, N deposition in the boreal forest biome accounted for only 0.7% of the global total increase. Compared with some forest biomes, some non-forest biomes, including cropland, grassland, shrubland and savanna, had more pronounced amounts and rates of increase in N deposition (Table S4).

The spatial distribution and increasing rate limit the fertilization effect of N deposition on the global C budget. Forest biomes are important for the global C budget, although the spatial disparity between the required N (for forest C assimilation and absorption) and increasing N deposition led to less significant increases in NPP and NEP. Increased N deposition in non-forest biomes did not contribute greatly to the global C budget.

### Influence of N saturation on the global C budget

**Figure 3:**
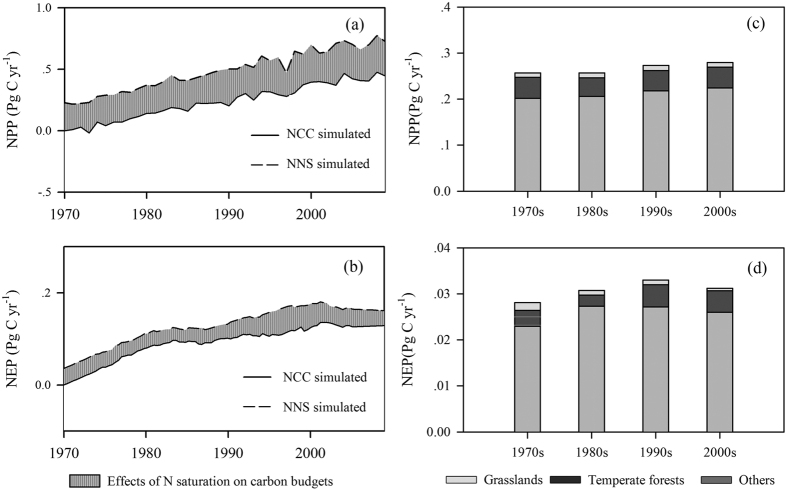
The impact of N saturation on the global C budget (NNS-NCC). (**a**) and (**b**) show the N-saturation effects on NPP and NEP in N-saturated regions. (**c**) and (**d**) show the N-saturation effects of the vegetation types.

To evaluate the impact of N saturation on the global C budget, we designed a comparison experiment between the simulation scenarios NNS (No Nitrogen Saturation) and NCC scenarios. In the NNS scenario, the effects of N saturation were removed by modifying the equations for three C-N coupling modifiers, *K*_P_, *K*_1_ and *K*_M_, which were set to 1.0 when N deposition exceeded the N critical load. The results of a comparison between the NNS and NCC scenarios are shown in Fig. 3. N saturation caused the terrestrial ecosystem NPP and NEP to decline. N saturation resulted in an NPP decrease of 0.26 Pg C yr^−1^ and an NEP decrease of 0.03 Pg C yr^−1^, accounting for 0.5% and 1.3% of the annual average global total NPP and NEP, respectively, between 1970 and 2009.

The negative effects of N saturation on the global C budget can be primarily attributed to temperate forests and grasslands. In temperate forests, NPP and NEP were reduced by 0.04 Pg C yr^−1^ and 0.005 Pg C yr^−1^, respectively. In grasslands, the NPP and NEP decreased by 0.2 Pg C yr^−1^ and 0.025 Pg C yr^−1^, respectively. Along with rapid increases in N deposition, the harm caused by N saturation was substantial in grasslands, which led to substantial decreases in NPP and NEP. These two vegetation types responded primarily to the change in N saturation over the past 40 years.

### Sensitivity of the C budget to changes in N deposition

**Figure 4:**
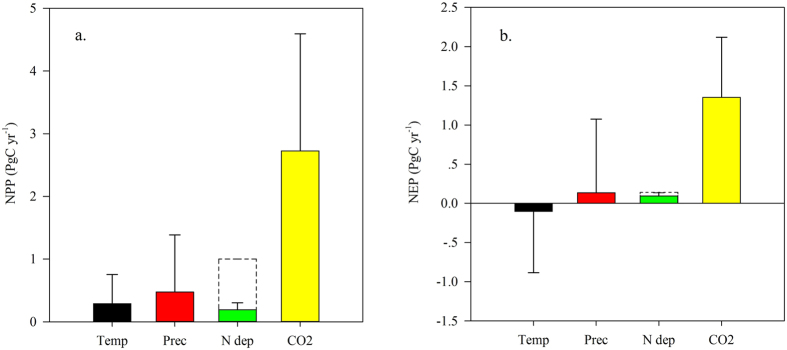
The contributions of changes in temperature, precipitation, N deposition and CO_2_ to the global C budget under global change. (**a**) shows the contributions of driving factors to NPP; (**b**) shows the contributions of driving factors to NEP. (Temp is temperature, Prec is precipitation, N dep is N deposition). The dashed boxes in (**a**) and (**b**) indicate the contributions of N deposition to NPP and NEP, respectively, when N saturation effects are not considered.

N deposition and N saturation have well-defined impacts on the global C budget. To compare the influence of N deposition to other global change factors (e.g., CO_2_, temperature and precipitation), five comparison simulations (i.e., the NCC, CNC (CO_2_ No Change), NNC, TNC (Temperate No Change) and PNC (Precipitation No Change) scenarios were performed using different driving factor settings, details are listed in Table 1. A summary of the relative contributions of the different driving factors is provided in Fig. 4.

Our results indicate that elevated CO_2_ promoted NPP and NEP. With increasing CO_2_ levels, NPP and NEP increased by 2.7 Pg C yr^−1^ (5.4% of the global total) and 1.4 Pg C yr^−1^ (51.0% of the global total), respectively. Rising atmospheric CO_2_ accelerates the photosynthetic rate by increasing intercellular CO_2_ and decreasing stomatal conductance, with significant effects on the global C budget^40^. Increases in the global temperature promote NPP but inhibit NEP, and changes in precipitation promote both NPP and NEP. However, due to their large standard deviations, the effects of temperature and precipitation on the global C budget are highly uncertain^41^.

Compared with the effects of temperature, precipitation and CO_2_, the contributions of N deposition to NPP and NEP were less significant. This does not mean that N deposition is not sensitive to the global C budget; N saturation weakens the contribution of N deposition. Without considering N saturation, the impact of N deposition on NPP was markedly greater than the impact of temperature or precipitation, and the impact of N deposition on NEP was nearly equivalent to the impact of temperature and precipitation. N saturation, in addition to the heterogeneous spatial distribution of N deposition, is the most important factor limiting the positive effects of N deposition on the global C budget.

## Discussion

Ecosystem N saturation is a complex process characterized by soil mineralization and nitrification processes, nitrate leaching from water, the C:N ratio of vegetation, and other factors. In this study, the N critical load was used to determine ecosystem N saturation. The concept of the N critical load was used to establish the N input level that an ecosystem can tolerate without significant harmful effects^42^, which can be calculated using empirical methods or by soil process models. Compared with the methods used for site observations (i.e., N leaching or isotopic indicators), N critical loads reflect the effects of N deposition on the ecosystem at a regional scale. Furthermore, the N critical load has been used by many researchers for different vegetation types around the world, particularly in regions enhanced by N deposition. Therefore, it is reasonable to use the N critical load as an indicator of N saturation.

The N saturation regions identified in our simulations have also been identified in previous studies of regional N saturation. N-saturated regions were typically characterized by dense population and industry. N saturation was most prevalent in temperate regions with strong anthropogenic N emissions. Consistent with the simulation results of the present study, N-saturation regions in U.S. and European forests have been reported in previous studies. More than 25% of European forests are N-saturated^19^. In the northeastern US, results from several observation sites have shown that N saturation is a frequently occurring phenomenon^22^. In some East Asian forest regions, N deposition was higher than in Europe and the US, although N saturation did not occur. Some researchers believe that despite the high levels of N deposition in these forest regions, the ecosystem remains N-deficient due to the short history of elevated N deposition^43^.

**Figure 5:**
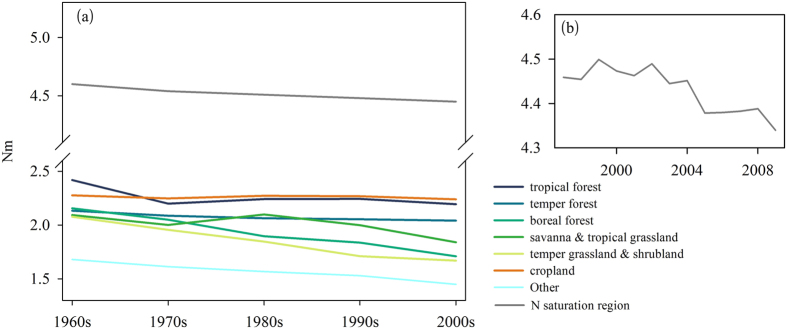
The change of *N*_M_ (soil mineral N) in biomes and N-saturation regions. (**a**) shows the average change of *N*_M_ from 1960 to 2009 in biomes and N-saturation regions; (**b**) shows the average change of *N*_M_ from 2000 to 2009 in N-saturation regions.

N deposition is important to the effects of elevated CO_2_ on C budgets as it supplies more available N in the soil. In almost all biomes, soil mineral N declined under elevated CO_2_ (Fig. 5a). More rapid decreases in soil mineral N were found in boreal forest, grassland and shrubland biomes where N deposition is lower. Smaller decreases were observed in regions that are strongly affected by the increase in N deposition, such as temperate and tropical forest. If N deposition had not increased over the past few decades, soil mineral N would have declined more rapidly in temperate and tropical forests. The increased N deposition meets the increasing N demand of CO_2_ fertilization. Simultaneous increases in atmospheric CO_2_ and N deposition are significant for global C cycling because they promote increased C assimilation and absorption. However, the current effects of N saturation and the spatial distribution of N deposition have limited the contribution of N deposition to the global C budget.

N saturation was also affected by increasing atmospheric CO_2_ concentration. Elevated CO_2_ affected not only global C cycles but also global N turnover. As shown in Fig. 5b, simulated soil mineral N declined, even in N-saturated regions. This decline can be mainly attributed to elevated CO_2_, which increases plant N demand and decreases N turnover. According to our simulations, the effect of CO_2_ on N turnover is larger than the effect of N deposition, although N deposition has a strong effect on N turnover in N-saturation regions (Table 2). In some CO_2_-enrichment experiments and field observation studies, elevated CO_2_ increased N uptake in the ecosystem^44^,^45^,^46^. From a global perspective, over the past few decades, the rising atmospheric CO_2_ concentration has been the main factor driving increasing global C sequestration. In such a process, increased soil mineral N is absorbed into vegetation biomass, and N retention in soils declines at elevated CO_2_ levels. Although ecosystems have several mechanisms to compensate for N deficiency, such as strengthening N fixation and enhancing decomposition, soil mineral N may still decline, as reported in FACE (Free-Air CO_2_ Enrichment) experiments^44^,^47^. Therefore, along with the soil mineral N decline resulting from elevated CO_2_, the ecosystem N saturation was changed.

In the future, the negative effects of N saturation on the global C balance will continue to be complex. Trade-offs between the effects of rising N deposition and elevated CO_2_ fertilization will determine the fate of soil mineral N. Rapidly increasing CO_2_ levels increase the severity of the effects of N limitation; some regions of N saturation will become more sensitive to N limitation. However, if N deposition increases rapidly, the distribution of N saturation will be extended, and the negative effects of N saturation will be enhanced.

Our research has some limitations. A major limitation of this study involves the determination of the soil mineral N level when N saturation occurred. We used the N critical load to represent the soil mineral N when N saturation occurs in the IBIS model. However, due to the limited number of observational and experimental studies, a complete global distribution of N critical loads could not be determined. Using the average N critical loads for different vegetation types to determine N saturation contributes uncertainty to the estimation result. The Markov chain Monte Carlo method is a powerful way to determine model parameters and could also contribute uncertainty to the estimation. With the increasing availability of N-saturation observation data, the Markov chain Monte Carlo method could be used to estimate the magnitude of the model uncertainty in the future.

## Methods

### IBIS model description

The original IBIS model^48^,^49^ tracks soil N along with SOC (Soil Organic Carbon), although there are no soil N controls based on vegetation productivity. Liu *et al*.^50^ incorporated a largely complete N cycle module into the IBIS model, which focuses on new N feedback controls for both aboveground C assimilation and belowground SOC decomposition while imposing a balanced N budget requirement. Several new control factors were introduced into the IBIS model to control the C-N cycling process, such as *K*_P_, *K*_1_ and *K*_M_, which are modifiers of plant biomass construction, soil N immobilization and soil organic matter mineralization, respectively. More details about the N cycle module are provided in Supplemental Information SI.1.

### Modification of the IBIS model

**Figure 6:**
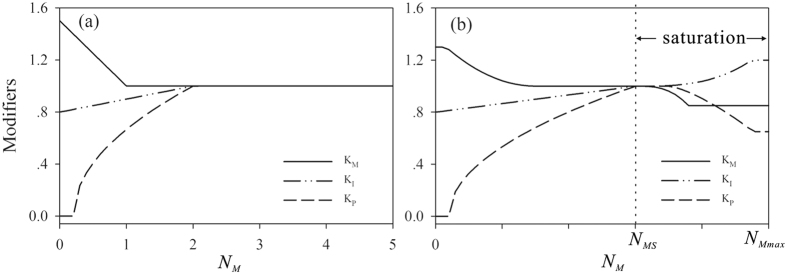
The conceptual N-control modifiers in IBIS. (**a**) modifiers used in IBIS by Liu *et al*.^50^; (**b**) modifiers used in IBIS in this study. *N*_Mmax_ is the maximum N available in the soil, *N*_MS_ is the soil mineral N when N saturation occurs, and *N*_M_ is the soil mineral N.

The modification to IBIS put forth by Liu *et al*.^50^ focused on the response of an ecosystem to increased soil N, and it did not account for the effect of excessive soil N. The three important N cycle control modifiers *K*_P_, *K*_1_ and *K*_M_ were utilized by Liu *et al*.^50^. When *N*_M_ (soil mineral N) exceeds 2 g m^−2^, *K*_P_, *K*_1_ and *K*_M_ are fixed at 1 (Fig. 6a), indicating that the ecosystem contains sufficient N to support plant growth and will not respond to additional N input. The work of Liu *et al*.^50^ focused on Canadian boreal forests, where the soil N deficit limits forest growth. However, on a global scale, N saturation caused by human fertilization and N deposition is significant and should not be ignored. To simulate ecosystem responses to N saturation, we further modified IBIS based on recent findings to include three aspects of the N saturation effect on the C budget: C assimilation, C allocation and SOC decomposition.

Many studies have focused on the positive effects of N addition on C assimilation in N-limited ecosystems^3^,^51^. Only a few studies have focused on the effect of N saturation on C assimilation in long-term N fertilization experiments. The results of the N saturation experiments conducted by Magill *et al*.^23^ indicated that following 15 years of N addition, the forest NPP markedly declined. However, this decline varied among forest species. Long-term N addition resulted in a 37% decline in the NPP of Korean pine forest, whereas in hardwood forest, the NPP decline was 6% following partial treatments. Mo *et al*.^18^ found that the most extreme N treatment caused a 10% decrease in the NPP in tropical forests in southern China. Based on these studies, we parameterized the maximum N saturation effect such that the NPP decreased by an average of 17.6%.

Previous studies have indicated that N saturation typically leads to an increase in C allocation to aboveground biomass and a decrease in C allocation to belowground biomass^18^,^25^,^52^. Litton *et al*.^53^ reviewed forest C allocation studies worldwide and found that when challenged with excessive N input, C allocation to roots decreases by 35% on average, whereas C allocation to leaves and wood increases by 10% and 25%, respectively.

In N-limited ecosystems where N deposition ranges from 0.5 to 1.0 g m^−2^ yr^−1^, additional N input promotes the decomposition of SOC^54^. However, in N-saturated ecosystems, greater N input has negative effects on SOC decomposition. Janssens *et al*.^25^ used meta-analysis methods to review the responses of SOC decomposition to rich N fertilization in 36 experiments conducted in forest ecosystems.

They found that SOC decomposition declined by 15% on average due to N saturation.

To incorporate the effect of N saturation on C assimilation, C allocation and SOC decomposition, we modified the *K*_P_, *K*_1_ and *K*_M_ factors in IBIS as Fig. 6b shows. The new equations used to calculate *K*_P_, *K*_1_, and *K*_M_ are as follows:


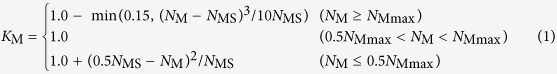



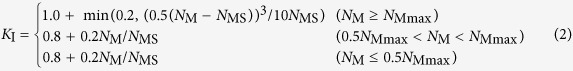



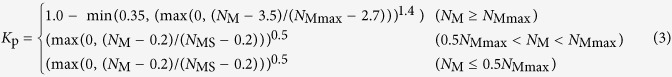


where *N*_Mmax_ is the maximum N available in the soil, *N*_MS_ is the soil mineral N when N saturation occurs, and *N*_M_ is the soil mineral N.

*N*_MS_ is the key parameter in equations (1), (2) and (3) and is related to the occurrence of N saturation. Many previous studies have used the N critical load to associate N deposition and ecosystem damage. Empirical N critical loads for the US, Europe and China can be found in the literature^55^,^56^,^57^. In this study, we used N critical loads to determine *N*_MS_ in the IBIS model. These results and classified N critical loads according to the putative vegetation types modelled in IBIS are shown in Table 3. The empirical N critical loads in other parts of the world were taken as the average value of the US, China and Europe.

In this study, empirical N critical loads were considered the maximum N deposition level resulting in no harm to the ecosystem. If N deposition exceeds this value, N saturation will occur. When N deposition reaches the N critical load, the soil mineral N content becomes important for calculating the N cycle parameters, including *K*_P_, *K*_1_ and *K*_M_. Thus, we set the N deposition level equal to the empirical N critical loads in the ecosystems detailed in Table 3; then, we set the key parameters (*K*_P_, *K*_1_ and *K*_M_) of the N cycles to 1. This means that there is no positive or negative effect of N control on the soil mineral N content when the N input reaches the maximum ecosystem demand. Thus, the soil mineral N critical load under N deposition is taken to be *N*_MS_ in the IBIS model.

### Driving data and model experiments

IBIS climate inputs include historical monthly precipitation and temperature data as well as the monthly average cloud fraction, wind speed, number of wet days per month, and relative humidity. All of these data were extracted from CRU (Climate Research Unit). The surface condition data consist of the vegetation cover fraction, initial biomass C, initial soil C, soil texture, and topography. Vegetation cover fractions were calculated based on the 300 m resolution GLOBCOVER 2009 map (http://www.esa.int/due/ionia/globcover). Initial biomass was derived based on Olson’s World Ecosystem database^58^, and soil C was derived from the Global Organic Soil Carbon and Nitrogen datasets^59^. The global atmospheric CO_2_ concentration was derived from ESRL (Earth System Research Laboratory) measurements obtained at NOAA (National Oceanic and Atmospheric Administration) and were used as the global average CO_2_ level (GLOBALVIEW-CO_2_, 2011).

Monthly N deposition data collected between 1970 and 2009 were estimated based on the NO_2_ column density associated with the output of the atmosphere chemistry transfer model MOZART (Model for Ozone and Related Chemical Tracers) using a previously reported method^60^.

Our simulation was divided into 3 stages. The first was the pre-heat stage from 1851 to 1900, where the average monthly climate data were used to drive the model to C equilibrium. N deposition at this stage was set to a baseline value of 0.05 g m^−2^ yr^−1^. The second stage was from 1901 to 1969. Actual monthly climate data were used to drive the model. The N deposition rate of 1970 was used at this stage. The third stage was from 1970 to 2009. In this stage, both the actual monthly climate data and monthly N deposition data were used to drive the model to simulate the global C budget.

To evaluate the effects of N deposition and other factors on global C budgets, six different simulations were used. The details are listed in Table 1. In the NCC scenario, all of the driving factors were dynamic; in the other five scenarios, one of the driving factors was held constant to determine the effect of each driving factor on C cycling. The NNS scenario is used to evaluate the N saturation effects on global C budgets. In this scenario, *K*_P_, *K*_1_ and *K*_M_ were set to 1 in N-saturated regions when N deposition exceeded the *N*_MS_.

### Model validation

Model outputs were validated against published literature and datasets^33^,^38^,^61^,^62^. Literature results and MODIS products were used to validate IBIS GPP (Gross Primary Productivity), NPP, and NEP. Our simulated GPP (122.3 ± 3.3 Pg C yr^−1^) is similar to the multiple-year average GPP of MTE (Model Tree Ensembles) (119 ± 6 Pg C yr^−1^) during the period 1980–2009, and the distribution of biases between the IBIS GPP and MTE GPP is reasonable (details shown in Supplemental Information SI.2.1). Based on a literature review, our simulated NPP is consistent with previous findings. From 1990 to 2009, the IBIS-simulated multi-year average NPP was 53.8 Pg C yr^−1^; this value is similar to the values of other models that consider C-N coupling and lower than those that include only a C cycling module. Our addition of the N saturation module in IBIS improves the simulated NPP, making it similar to the MODIS NPP (details in Supplemental Information SI.2.2). A global multi-year average NEP was generated via different methods to validate our simulated NEP (Table S3). The validation results show that the simulated NEP falls within the range of previous studies. The response of the ANPP (aboveground NPP) to N addition was examined based on datasets collected from N-addition experiments in grasslands and forests (Supplemental Information SI.2.4). Our simulated ANPP increased by 18 ± 13%, which is similar to the observation results (23 ± 9%). Thus, the sensitivity of N control to the global C budget in the IBIS model is reasonable.

## Additional Information

**How to cite this article**: Lu, X. *et al*. Simulated effects of nitrogen saturation on the global carbon budget using the IBIS model. *Sci. Rep.* **6**, 39173; doi: 10.1038/srep39173 (2016).

**Publisher’s note:** Springer Nature remains neutral with regard to jurisdictional claims in published maps and institutional affiliations.

## Supplementary Material

Supplementary Information

## Figures and Tables

**Table 1:** Design of the simulation experiments.

No.	Experiment	Full name	Temperature	Precipitation	CO_2_	N deposition
1	NCC	Nitrogen CO_2_ Climate	Change	Change	Change	Change
2	NNC	No Nitrogen Change	Change	Change	Change	1970
3	CNC	CO_2_ No Change	Change	Change	1970	Change
4	TNC	Temperate No Change	1970	Change	Change	Change
5	PNC	Precipitation No Change	Change	1970	Change	Change
6	NNS*	No N Saturation effect	Change	Change	Change	Change

^*^Modifier factors in the biogeochemical model are set to 1 when N saturation occurs.

**Table 2:** CO_2_ and N-deposition effects on N turnover (N in biomass is g N yr^−1^; N uptake is g N m^−2^ yr^−1^).

	NCC-CNC (CO_2_ effect)		NCC-NNC (N dep. effects)		NCC-NNS (N saturation effects)	
	N in biomass	N uptake	N in biomass	N uptake	N in biomass	N uptake
Tropical forest	3.5	0.4	0.8	0.3		
Temperate forest	1.4	0.3	0.2	0.01	0.6	0.17
Boreal forest	1.0	0.2	0.1	0.01		
Grassland	1.0	0.1	0.01	0.01	0.2	0.05

**Table 3:** N critical loads in vegetation types.

		Empirical critical loads of N (g N m^−2^ yr^−1^)			
Vegetation type		USA	China	Europe	Other
1	Tropical evergreen forest	0.9*	5.6	—	3.25
2	Tropical deciduous forest	0.9*	5.6	—	3.25
3	Temperate evergreen broadleaf forest	0.9	2.25	1.5	1.55
4	Temperate evergreen conifer forest	0.9	2.25	1.5	1.55
5	Temperate deciduous forest	0.9	2.25	1.5	1.55
6	Boreal evergreen forest	0.57	1.25	1.25	1.02
7	Boreal deciduous forest	0.57	1.25	1.25	1.02
8	Mixed forest	0.8**	2.9**	1.4**	1.7
9	Savanna	—	5	—	5
10	Grassland	—	1.25	1.45	1.35
11	Dense shrubland	0.47	0.92	—	0.69
12	Open shrubland	0.47	0.92	—	0.69
13	Tundra	0.20	0.75	0.4	0.45
14	Desert	0	1	0	1
15	Polar desert/rock/ice	0	1	0	1
	Reference	Pardo *et al*.^55^	Liu *et al*.^57^	Bobbink and Hettelingh^56^	***

^*^Since empirical data on nitrogen critical load (CL) were lacking for tropical forests in the US, we used the CL values of temperate forests in the US as the CL values of tropical forests.

^**^The CL value of mixed forest is the average CL value of different forests types in the US Europe and China.

^***^The CL values in other parts of the world are average values of the US, China and Europe.
