# Supplementary material for: Simulated effects of nitrogen saturation on the global carbon budget using the IBIS model
Source: Sci Rep. 2016 Dec 14;6:39173. doi: 10.1038/srep39173 (PMC5155240; doi:10.1038/srep39173)
Supplement: Supplementary Information [file srep39173-s1.pdf]

Simulated effects of nitrogen saturation on the global carbon budget using the IBIS model

Xuehe Lu[1,2], Hong Jiang[1,2*], Jinxun Liu[3], Xiuying Zhang[1,2], Jiaxin Jin[1,2], Qiuan Zhu[4], Zhen Zhang[1,2], Changhui Peng[4]

[1]Jiangsu Provincial Key Laboratory of Geographic Information Science and Technology, Xianlin Avenue 163, Nanjing 210093, China; [2]International Institute for Earth System Science, Nanjing University, Xianlin Avenue 163, Nanjing 210093, China; [3]USGS Western Geographic Science Center, Menlo Park, CA, 94025, USA; [4]State Key Laboratory of Soil Erosion and Dryland Farming on the Loess Plateau, Northwest A&F University, Yangling 712100, China

*Corresponding author: E-mail address: jianghong@nju.edu.cn Tel.: +86 25 89685969

Supplemental information

SI 1 N module in the IBIS model

The original IBIS (Integrated BIosphere Simulator)[1] has a very simple N (nitrogen) control on the NPP (Net Primary Productivity) calculation through a constant leaf N level. Liu *et al.*[2] incorporated a largely complete N cycle module into IBIS that includes the dynamic leaf N level and N controls on C (carbon) assimilation, C allocation and the C-N (Carbon-Nitrogen) cycle in soil[2].

Vegetation photosynthesis is characterized by the Farquhar equation in the IBIS model, and the maximum photosynthetic velocity is controlled by leaf-available N (equation (1)).

$$V_{\mathrm{m}} = (B_{\mathrm{Vmax}} / B_{\mathrm{L}}) \times V_{\mathrm{max}} \tag{1}$$

B_{Vmax} is the optimal C:N ratio for foliage, i.e., the ratio at which the maximum photosynthetic rate (V_{max}) occurs. B_{L} is the actual foliar C:N ratio, and V_{m} is the actual photosynthetic rate. If an N shortage exists, the actual foliar C:N ratio (B_{L}) will increase and V_{max} will decline. In the IBIS

model, this equation indicates the role of N feedbacks on photosynthesis.

A new control factor is used to adjust NPP accumulation in the IBIS model. The equation for K_{p} is shown in equation (2) below, and its influence on NPP accumulation is described in equation (3).

$$K_{\mathrm{p}} = \begin{cases} [(N_{\mathrm{M}} - 0.2) / (N_{\mathrm{M\,max}} - N_{\mathrm{M}})]^{0.5} & (N_{\mathrm{M}} < N_{\mathrm{Mmax}}) \\ 1 & (N_{\mathrm{M}} \geq N_{\mathrm{Mmax}}) \end{cases} \tag{2}$$

K_{p} is a control factor, N_{M} is the available N in the soil, and N_{Mmax} is the maximum available N in the soil, which was set to 2 g m^{-2} in the study of Liu *et al.*[2]

$$NPP_{\mathrm{d}} = NPP_{\mathrm{d}} \times (1.0 - R_{\mathrm{g}}) \times K_{\mathrm{p}} \tag{3}$$

NPP_{d} is the daily NPP, and R_{g} is the growth respiration ratio. K_{p} is used to adjust the effect of respiration on NPP. When the soil N level is low, even leaf photosynthesis permits a high NPP, and the actual NPP will be low due to modification by K_{p}. K_{p} is also used to regulate the carbon allocation of vegetation (equation (4)). In N-limited ecosystems, K_{p} limits respiration and stimulates greater C allocation to roots. Thus, vegetation can promote root growth and absorb more N in the soil to adapt to N limitation.

$$\begin{aligned} A_{\mathrm{root}} &= A_{\mathrm{root_{max}}} - K_{\mathrm{p}} \times (A_{\mathrm{root_{max}}} - A_{\mathrm{root_{min}}}) \\ A_{\mathrm{leaf}} &= A_{\mathrm{leaf\text{-}root}} - A_{\mathrm{root}} \\ A_{\mathrm{wood}} &= 1 - A_{\mathrm{leaf\text{-}root}} \end{aligned} \tag{4}$$

Here, A_{root}, A_{leaf} and A_{wood} represent the C allocation ratio in the root, leaf and wood components, respectively. $A_{\mathrm{root_{max}}}$ and $A_{\mathrm{root_{min}}}$ are the maximum and minimum C allocation ratios of the roots, respectively. $A_{\mathrm{leaf\text{-}root}}$ is the sum of the root and leaf allocation ratios.

The role of the N feedback in soil C decomposition is expressed in equation (5):

$$C_{\mathrm{x}} = \begin{cases} d_{\mathrm{s}} y_{\mathrm{ij}} K_{\mathrm{i}} P K_{\mathrm{I}} C_{\mathrm{i}} & \delta_{ij} \leq 0 \\ d_{\mathrm{s}} y_{\mathrm{ij}} K_{\mathrm{i}} P K_{\mathrm{M}} C_{\mathrm{i}} & \delta_{ij} > 0 \end{cases} \tag{5}$$

C_{x} is the actual decomposition rate and is controlled by certain control factors. Moreover, d_{s}, y_{ij},

K_i , and C_i belong to the original IBIS model, of which d_s is the coefficient representing soil moisture and temperature effects on decomposition, C_i is the C pool, y_{ij} is the yield coefficient when C_i is transferred from source i to target j , and K_i is the fixed base decomposition rate of each SOC (Soil Organic Carbon) pool. Moreover, P is a factor that controls SOC decomposition according to the soil C situation (i.e., the priming effect). δ_{ij} is the identifier that indicates whether a process absorbs or releases N. K_I and K_M are two new factors controlled by the available N in the soil (equation (6)):

$$K_M = \begin{cases} 1.0-(N_M - N_{Mmax})/N_{Mmax} & (N_M \geq N_{Mmax}) \\ 1.0 & (0.5N_{Mmax} < N_M < N_{Mmax}) \\ 1.0+(0.5N_{Mmax} - N_M)/N_{Mmax} & (N_M \leq 0.5N_{Mmax}) \end{cases}$$
$$K_I = 0.8 + 0.2N_M / N_{Mmax} \tag{6}$$

N_{Mmax} is the maximum available mineral N in the soil (2 g N m^{-2}) that allows N limitation to occur. N_M is the actual available mineral N in the soil.

SI. 2 Model validation

SI. 2.1 Validation of GPP

According to IPCC AR4 (Intergovernmental Panel on Climate Change Fourth Assessment Report)[3], the GPP (Gross Primary Productivity) of the terrestrial ecosystem is 120 Pg C yr^{-1}. Beer *et al.*[4] determined that the global terrestrial ecosystem GPP is 123±8 Pg C yr^{-1} using global FLUXNET data. Jung *et al.*[5] used global FLUXNET data and MTEs (Model Tree Ensembles) to study the global C cycle and determined that the global terrestrial ecosystem GPP is 119±6 Pg C yr^{-1}. Our simulated average terrestrial ecosystem GPP is 122.3±3.3 Pg C yr^{-1} (between 1980 and 2000), which is very close to the values reported in previous studies.

A comparison of the simulated annual GPP from IBIS and MTE is presented in Figure S1. The multiple-year average IBIS GPP is similar to the MTE GPP, although the trends in the IBIS GPP and MTE GPP differ after 1995. It is generally agreed that the MTE should not be used as a benchmark for GPP trends[6]. One reason is that the CO_2 fertilization effect was not considered in the

MTE. Another reason is that the flux tower sites used in the MTE are mainly distributed in northern temperate regions, whereas tropical ecosystems largely drive the inter-annual variability in the C cycle[7]. Other modelled GPP trends have been summarized by Anav *et al.*[6] The inter-annual trend in GPP ranged from 0.28 to 0.62 Pg C yr^{-2} between 1990 and 2010, which is comparable to the trend in the IBIS GPP (i.e., 0.39 Pg C yr^{-2}) during the same period (1990-2009). Thus, the IBIS GPP is similar to previous findings in terms of both magnitude and trend.

The spatial differences between the IBIS GPP and MTE GPP are shown in Figure S2. In most grids, the IBIS GPP and MTE GPP are consistent. The average errors were less than ±20% in most tropical, temporal and boreal forests. The largest biases occurred in the southwestern U.S., southern South America and central Asia. These bias zones are mainly dry tropic regions (10–30°S and 10–30°N), and the dominant vegetation types are desert and open shrubland, which lack flux tower measurements. Thus, a larger sampling uncertainty is associated with the MTE GPP in these zones than in other zones[8]. Meanwhile, according to a review by Anav *et al.*[6], the bias in GPP between process-based models and MTE is approximately ±50% for the dry tropics. Thus, when comparing the IBIS GPP with the MTE GPP, the large biases shown in the dry tropics are not unexpected.

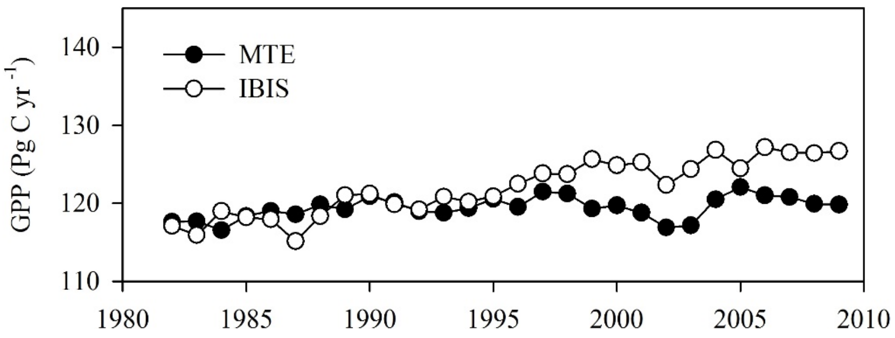

Figure S1 Simulated inner-annual GPP comparison with the MTE GPP.

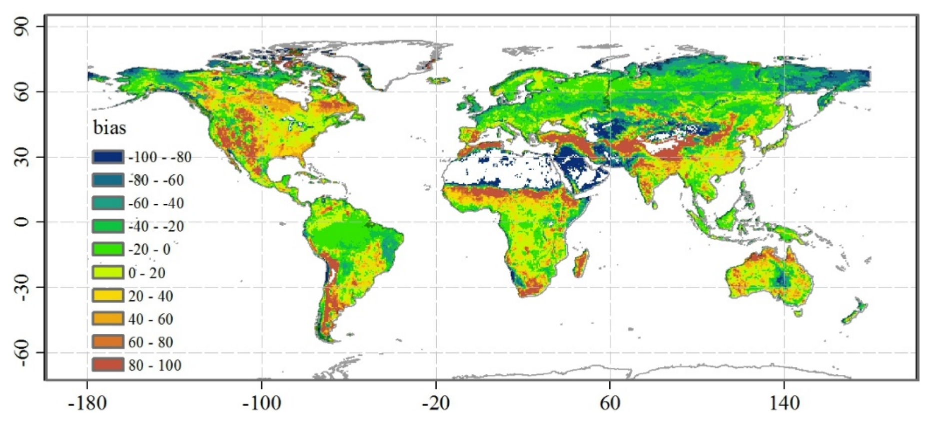

Figure S2. Spatial distribution of the differences between the IBIS GPP and MTE GPP (%). The map was generated using ArcGIS 10.0 software (https://www.arcgis.com/).

We also compared our GPP with the GPP values obtained by Beer *et al.*[4] and Saugier *et al.*[9] in different biomes (Table S1). The distribution of global biomes is based on the work of Roy *et al.*[10] and Griggs and Noguer[11]. In addition, cropland has been incorporated into global biomes, which is extracted from the MODIS (MODerate resolution Imaging Spectroradiometer) land cover product. The IBIS-simulated forest GPP value is higher than Beer's value but similar to Saugier's value. Compared with Beer's GPP, the IBIS GPP is higher in tropical and temperate forests. However, in tropical savanna and grasslands, the IBIS GPP is lower than in previous studies. In other biomes, the IBIS GPP results are similar to those of other studies.

Table S1. GPP for different biomes around the world (1980-2000; unit: Pg C yr^{-1}).

Vegetation type	GPP	GPP=2NPP	GPP
Reference	Beer *et al.*[4]	Saugier *et al.*[9]	IBIS
Tropical forest	40.8	43.8	43.1
Temperate forest	9.9	16.2	12.3
Boreal forest	8.3	5.2	8.6
Tropical savanna & grassland	31.3	29.8	24.8
Temperate grassland & shrubland	8.5	14	7.5
Desert	6.4	7	8.0
Tundra	1.6	1	1.3
Other	14.8	8.2	16.3
Total	121.7	125.2	121.9.

SI. 2.2 Validation of the NPP

We compiled recent global terrestrial NPP results covering the past 30 years (Table S2). Using meta-analysis, Ito *et al.* [12] summarized 251 studies of global NPP spanning the period from 1862 to 2011 and found that the global multi-year average NPP was 56.2±14.3 Pg C yr^{-1}. Our simulated average NPP is 53.8 Pg C yr^{-1} from 1980 to 2009, which is very close to Ito's value. Similar to the NPP of other models that consider C-N coupling, our simulated NPP is lower than the model results that include only a C cycling module. In general, our simulation results fall within the range of previous studies.

In addition to using data from published papers to validate the global total NPP, we used the MODIS NPP product to examine the performance of the IBIS N-saturation module. Without considering N saturation, IBIS overestimates the NPP compared with the MODIS NPP. However, with the N-saturation module, the IBIS NPP is more consistent with the MODIS NPP (Figure S3).

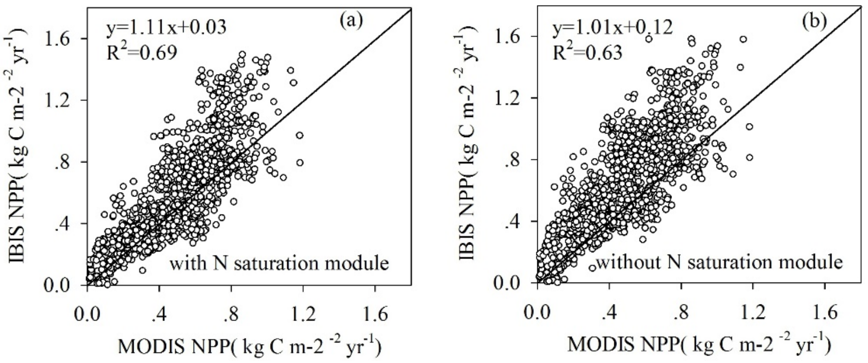

Figure S3. Comparison of the simulated IBIS NPP and the MODIS NPP in N-saturated regions. (a) IBIS NPP simulated with an N-saturation module; (b) IBIS NPP simulated without an N-saturation module.

Table S2. Validation of the global NPP.

Reference	Method	NPP (Pg C yr^{-1})	Time range	This study (Pg C yr^{-1})
Houghton *et al.*[13]	Review	60	IPCC AR3	
Schlesinger[14]	Land surface process model	60		
Cramer *et al.*[15]	Land surface process model	45-60	1901-1998	46.6-57.4
Gruber *et al.*[16]	Review	57		
Zhao *et al.*[17]	Remote sensing	56.02	2001-2003	54.8
Thornton *et al.*[18]	Land surface process model (C-N)	44.7	1974-2000	52.7
Thomas *et al.*[19]	Land surface process model (C-N)	50	1985-2009	54.2
Zaehle[20]	Land surface process model (C-N)	53.1	2001-2010	≈55.8

SI. 2.3 Validation of the NEP

In this study, we collected global NEP (Net Ecosystem Productivity) results evaluated by different methods to validate our simulated NEP (Table S3). The IBIS-simulated NEP average was found to be 2.5 Pg C yr^{-1} from 1980 to 2000, which is within a reasonable range. A previous study also

showed that the NEP result of a C-N coupling model is similar to that of a C model, although the spatial distribution of NEP and the sensitivity of the terrestrial C balance to its driving factors are substantially altered by N dynamics[21].

Table S3. Validation of the NEP for a global terrestrial ecosystem.

Reference	Method	NEP (Pg C yr^{-1})	Time range	IBIS (Pg C yr^{-1})
Ciais *et al.*[22]	Atmospheric model	2	1985-1995	2.1
Cramer *et al.*[15]	6 DGVM[a] models	1.4-3.8	1901-1998	1.4±2.3
Richardson *et al.*[23]	FLUXENT model	2.6		
Tans[24]	Atmospheric model	1.5-3.0	1950-1999	1.9±1.2
Potter *et al.*[25]	NASA[b]-CASA[c] model	2.1	1982-1998	2.1
Pan *et al.*[26]	Inventory & model	2.4	1990-2007	2.63
Denman *et al.*[7]	Atmospheric model	2.6±1	IPCC AR4	
Zaehle *et al.*[21]	Land surface process model (C-N)	2.4	1990-1999	2.6
Thornton *et al.*[18]	Land surface process model (C-N)	1.24	1974-2000	2.3

[a] DGVM, Dynamic Global Vegetation Model

[b] NASA, National Aeronautics and Space Administration

[c] CASA, Carnegie-Ames-Stanford Approach

SI. 2.4 Validation of the N-cycling module

To validate the N control on C cycling, we used the observations of aboveground NPP responses to N addition in forests and grasslands. For forests, Thomas *et al.*[19] reviewed 40 N-addition experiments in which the N-addition rates ranged from 0.9 to 15.0 g N m^{-2} yr^{-1} over 2-30 years[19]. For grasslands, LeBauer and Treseder[27] reviewed 39 N-addition experiments around the world. We chose 37 experimental results because some of the studies were conducted on small islands that were not included in our simulations. The locations of the N-addition experiments are shown in Figure S4a.

We used the percentage change in aboveground growth between N-addition experiments and N-control baseline experiments to compare measured N responses with model simulations. Model simulations were performed for each grid cell containing a field experiment site. The modelled amount of added N was the same as in the field experiments. To facilitate the comparison, we used

the simulation strategy developed by Thomas *et al.*[19] The modelled aboveground NPP (ANPP) was calculated for the same time frame as the field study, and the N addition began after 1985.

The N control of C cycling has been studied for forests but not for grassland areas according to a comparison with observation results. The rate of forest ANPP increased by 23±9% on average in response to N fertilization in the field experiments. IBIS-simulated ANPP increased by 18±13%, which is similar to the empirical results (Figure S4b). However, in grassland areas, the rate of ANPP increase observed at field sites averaged 62±15%, whereas the IBIS-simulated ANPP increase was 35±25% (Figure S4c).

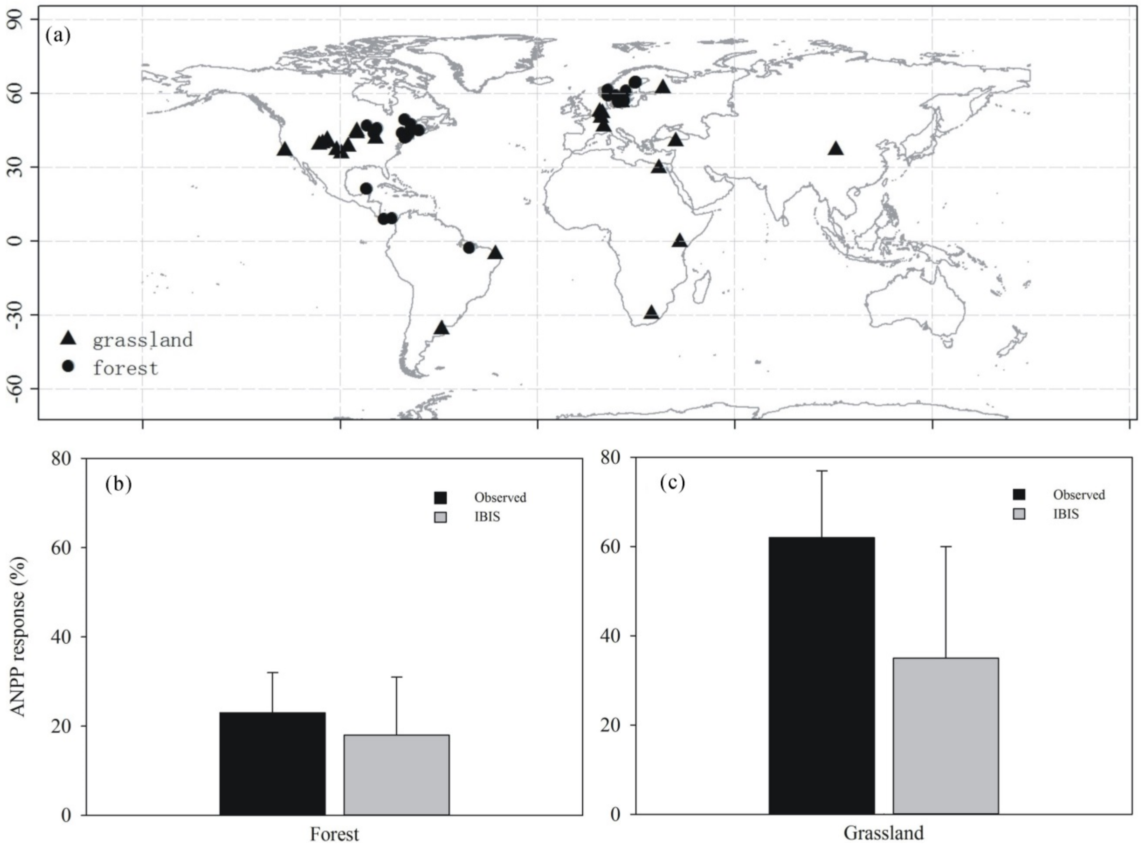

Figure S4. Response to N fertilization in forests and grasslands. (a) A map of N-fertilization experiments; (b) and (c) comparisons of the response to N fertilization according to observation and IBIS simulation. The maps were generated using ArcGIS 10.0 software (https://www.arcgis.com/) and SigmaPlot version 12.0, from Systat Software, Inc., San Jose California USA (https://www.systatsoftware.com).

SI. 3 NPP and NEP changes over 40 years

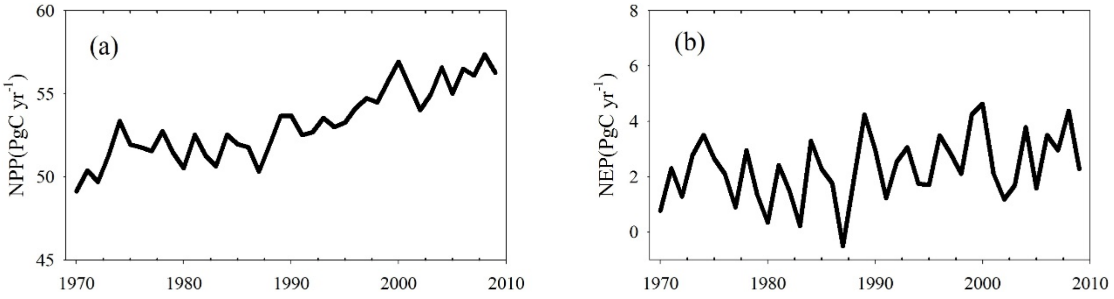

Figure S5. Historical NPP and NEP changes.

SI. 4 N deposition in different biomes

Table S4 The change of N deposition in different biomes

Biome	Total N deposition		Increasing percentage in total N deposition increasing (%)
	1970	2000	
Cropland	9.25	14.47	33.71
Tropical forest	5.05	9.07	25.94
Temperate forest	7.73	7.49	11.31
Boreal forest	2.67	2.77	0.66
Tropical savanna and grassland	4.89	6.27	8.93
Temperate grasslands and shrubland	2.2	3.14	6.1
Other	5.09	7.15	13.33
Total	34.90	50.37	100

References

1. Foley, J. A. *et al*. An integrated biosphere model of land surface processes, terrestrial carbon balance, and vegetation dynamics. *Global Biogeochem. Cycles* **10**, 603–628 (1996).
2. Liu, J., Price, D. T. & Chen, J. M. Nitrogen controls on ecosystem carbon sequestration: A model implementation and application to Saskatchewan, Canada. *Ecol. Model.* **186,** 178–195 (2005).
3. Solomon, S. *Climate change 2007: contribution of working group I to the fourth assessment report of the intergovernmental panel on climate change* (Cambridge University Press, 2007).
4. Beer, C. *et al.* Terrestrial gross carbon dioxide uptake: global distribution and covariation with climate. *Science* **329,** 834–838 (2010).
5. Jung, M. *et al.* Global patterns of land-atmosphere fluxes of carbon dioxide, latent heat, and sensible heat derived from eddy covariance, satellite, and meteorological observations. *J. Geophys. Res. Biogeo.* **116,** 245–255 (2011).
6. Anav, A. *et al.* Spatiotemporal patterns of terrestrial gross primary production: a review. *Rev. Geophys.* **53,** 785–818 (2015).
7. Denman, K. L. *et al.* Couplings between changes in the climate system and biogeochemistry. *Lawrn Brly Naonal Laboraory,* **2007,** 499–587 (2005).

8. Piao, S. *et al.* Evaluation of terrestrial carbon cycle models for their response to climate variability and to CO_2 trends. *Glob. Change Biol.* **19,** 2117–2132 (2013).
9. Saugier, B., Roy, J. & Mooney, H. A. Estimations of global terrestrial productivity: converging towards a single number? *Terr. Glob. Product.*, 543–558 (2001). doi: 10.1016 /B978-012505290-0/50024-7.
10. Roy, J., Saugier, B., Mooney, H. A. & McNaughton, S. J. Terrestrial global productivity. *Austral Ecol.* **27,** 584–585 (2002). doi: 10.1016/B978-012505290-0/50007-7.
11. Griggs, D. J. & Noguer, M. Climate change 2001: the scientific basis. Contribution of working group I to the third assessment report of the intergovernmental panel on climate change. *Weather* **57,** 267–269 (2002).
12. Ito A. A historical meta-analysis of global terrestrial net primary productivity: are estimates converging?. *Glob. Change Biol.* **17,** 3161–3175 (2011).
13. Houghton, J. T. Climate change 2001: the scientific basis. *Neth. J. Geosci.* **87,** 197-199 (2001).
14. Schlesinger, W. & Bernhardt, E. S. *The biosphere: the carbon cycle of terrestrial ecosystems in biogeochemistry: an analysis of global change* 3rd edn (ed. Schlesinger, W. H. & Bernhardt, E. S.) 135–172 (Academic Press, 2013).
15. Cramer, W. *et al.* Global response of terrestrial ecosystem structure and function to CO_2 and climate change: results from six dynamic global vegetation models. *Glob. Change Biol.* **7,** 357–373 (2001).
16. Gruber, N. *et al.* The vulnerability of the carbon cycle in the 21st century: an assessment of carbon-climate-human interactions in *The global carbon cycle: integrating humans, climate, and the natural world***,** 45–76 (Island Press, 2004).
17. Zhao, M., Heinsch, F. A., Nemani, R. R. & Running, S. W. Improvements of the MODIS terrestrial gross and net primary production global data set. *Remote Sens. Environ.* **95,** 164–176 (2005).
18. Thornton, P. E., Lamarque, J. F., Rosenbloom, N. A. & Mahowald, N. M. Influence of carbon-nitrogen cycle coupling on land model response to CO_2 fertilization and climate variability. *Global Biogeochem. Cycles* **21**, n/a–n/a (2007).
19. Thomas, R. Q., Zaehle, S., Templer, P. H. & Goodale, C. L. Global patterns of nitrogen limitation: confronting two global biogeochemical models with observations. *Glob. Change Biol.* **19,** 2986–2998 (2013).
20. Zaehle, S. Terrestrial nitrogen-carbon cycle interactions at the global scale. *Philos. Trans. R. Soc. Lond., B, Biol. Sci.* **368,** 125–134 (2013).
21. Zaehle, S. *et al.* Carbon and nitrogen cycle dynamics in the O-CN land surface model: 2. Role of the nitrogen cycle in the historical terrestrial carbon balance. *Global Biogeochem. Cycles***24,** 1–14 (2010).
22. Ciais, P., Peylin, P. & Bousquet, P. Regional biospheric carbon fluxes as inferred from atmospheric CO_2 measurements. *Ecol. Appl.* **10,** 1574–1589 (2000).
23. Richardson, A. D. *et al.* A multi-site analysis of random error in tower-based measurements of carbon and energy fluxes. *Agr. Forest Meteorol.* **136,** 1–18 (2006).
24. Tans, P. An accounting of the observed increase in oceanic and atmospheric CO_2 and the outlook for the future. *Oceanography* **22,** 26–35 (2009).
25. Potter, C. Continental-scale comparisons of terrestrial carbon sinks estimated from satellite data and ecosystem modeling 1982-1998. *Glob. Planet. Change* **39,** 201–213 (2003).

26. Pan, Y. *et al.* A large and persistent carbon sink in the worldrbon sinks. *Science* **333,** 988–993 (2011).
27. LeBauer, D. S. & Treseder, K. K. Nitrogen limitation of net primary productivity in terrestrial ecosystems is globally distributed. *Ecology* **89,** 371–379 (2008).
